# Bayesian spatio-temporal modeling for policy evaluation: Sensitivity of policy effect estimates in the context of COVID-19 stay-at-home orders

**DOI:** 10.1371/journal.pone.0339196

**Published:** 2026-02-10

**Authors:** Pyung Kim, Sunghye Choi, Dohyeong Kim, Chang-Kil Lee

**Affiliations:** 1 Center for Spatial Studies and Data Science, Department of Geography, University of California, Santa Barbara, Santa Barbara, California, United States of America,; 2 School of Economic, Political and Policy Sciences, The University of Texas at Dallas, Dallas, Texas, United States of America; 3 Department of Urban Policy and Administration, Incheon National University, Incheon, South Korea; Menzies School of Health Research: Charles Darwin University, AUSTRALIA

## Abstract

This study applies a Bayesian spatio-temporal model to demonstrate the sensitivity of policy effect estimates to spatial and temporal structure, using COVID-19 stay-at-home orders as a case study. Unlike conventional approaches, this framework accounts for geographic spillovers, temporal dependence, and space–time interaction, all of which are central to policy effect evaluation in heterogeneous settings. Implemented via Integrated Nested Laplace Approximation (INLA), the model also accommodates missing data and supports inference in high-dimensional contexts. Using Google mobility data and policy information from the Oxford COVID-19 Tracker, we estimate four models of increasing complexity: OLS, spatial, temporal, and spatio-temporal. While simpler models suggest substantial reductions in workplace and residential mobility, these effects become statistically insignificant once spatio-temporal interactions are incorporated. This pattern indicates that earlier studies may have overstated policy effects by overlooking spatio-temporal heterogeneity. Our findings demonstrate the importance of spatio-temporal modeling for policy evaluation, particularly when working with large-scale, incomplete, and unevenly distributed data.

## 1. Introduction

Health policies unfold across geographic and temporal dimensions, yet their impacts are rarely uniform across space or stable over time [1,2]. Nevertheless, conventional evaluation methods often treat interventions as static and geographically uniform shocks, overlooking how policy effects vary by location, period, and, most importantly, through their interaction. Adding to this complexity, real-world datasets used in policy evaluation frequently suffer from missing or uneven coverage across regions and timeframes [3]. Such missingness is often non-random and systematically related to key outcomes, introducing bias and undermining the credibility of findings. These dual challenges, spatio-temporal heterogeneity and data sparsity, demand analytic approaches that can jointly model spatial and temporal variation while rigorously addressing uncertainty due to incomplete data.

Bayesian spatio-temporal modeling offers a robust solution to these issues. By explicitly capturing interactions across space and time, Bayesian approaches enable more accurate and reliable inference under uncertainty, particularly when implemented using Integrated Nested Laplace Approximation (INLA) [4,5]. Unlike traditional frequentist methods, Bayesian models incorporate prior information, quantify uncertainty probabilistically, and perform well in high-dimensional or data-sparse settings [6,7]. The INLA framework, in particular, enables fast and flexible estimation of complex spatio-temporal structures, making it especially well-suited for health policy evaluation involving large, incomplete, or irregularly distributed data.

This study demonstrates the potential of Bayesian spatio-temporal modeling for evaluating the effects of health policies. We apply this framework to assess the impact of stay-at-home orders on human mobility across 1,422 counties in the southern United States between February 2020 and June 2021, a period marked by evolving public health guidance and changing behavioral patterns during the COVID-19 pandemic. By estimating and comparing four models of increasing complexity—OLS, spatial, temporal, and spatio-temporal—we examine how accounting for spatial and temporal structure alters inferences about policy effectiveness. Our findings reveal that policy effects identified in simpler models become statistically insignificant once spatio-temporal interactions are fully modeled, suggesting that previous studies may have overstated policy impacts by failing to account for spatiotemporal heterogeneity.

## 2. Bayesian spatio-temporal analysis for policy evaluation

### 2.1. Spatio-temporal model

Outcomes such as disease transmission, policy implementation, and human behavior are inherently shaped by both spatial and temporal dynamics. Effectively capturing the interaction between space and time is essential to understanding how outcomes evolve and diffuse across geographic contexts. During the COVID-19 pandemic, for example, human mobility was influenced not only by prior local mobility patterns (temporal dependence) but also by changes in neighboring regions (spatial dependence). A county’s mobility levels could decline following the implementation of stay-at-home orders in adjacent areas or a localized spike in cases, even without direct local policy changes, highlighting the importance of spatial spillovers and their temporal evolution.

Accounting for space-time interactions is essential not only for substantive interpretation but also for methodological validity. Modeling spatial or temporal effects in isolation risks conflating one with the other. For example, misattributing temporal trends to spatial variation or overlooking time-specific dynamics embedded in geographic patterns. Neglecting these interactions can result in biased estimates or false positives, such as mistakenly inferring policy diffusion or behavioral contagion where none exists. To mitigate these risks, recent research increasingly advocates for spatio-temporal modeling frameworks, which enhance the accuracy of dynamic process estimation and reduce the likelihood of model misspecification [8–10].

Spatio-temporal analysis typically integrates three core components: spatial effects, temporal effects, and their interaction [4,5]. Spatial analysis examines how outcomes vary across geographic units, identifying patterns of spatial dependence and clustering through metrics such as Moran’s I and Getis-Ord statistics. Temporal analysis captures changes over time, accounting for trends, seasonality, and autocorrelation using approaches like autoregressive (AR). By jointly modeling spatial and temporal structures, spatio-temporal analysis provides a unified framework for understanding dynamic processes that unfold across both space and time, allowing researchers to more accurately estimate evolving relationships and policy effects.

Formally, a general spatio-temporal model can be expressed as:


Y(s, t) ~ Distribution (μ(s,t), ϕ),

with μ(s,t)= α+X(s,t)β+τ(s)+ν(t)+δ(s,t),

Here, *Y*(*s, t*) represents the observed outcome at spatial location *s* and time *t*. The notation Distribution (μ(s,t), ϕ\)indicates that the observed data follow a probability distribution characterized by mean *µ*(*s, t*) and dispersion (or precision) parameter ϕ. The linear predictor *µ*(*s, t*) consists of a set of covariates *X* (*s, t*) with fixed effects *β*, a spatially structured random effects τ(s), a temporally structured random effects ν(t), and a space-time interaction term δ(s,t), which is often assumed to be independently and identically distributed.

This additive structure allows the model to flexibly capture both persistent and evolving spatial and temporal dynamics, as well as the interactions between them. By explicitly modeling each source of variation—spatial, temporal, and their interaction—the approach reduces the risk of omitted variable bias and confounding, which are common causes of model misspecification. For example, if temporal trends differ systematically across geographic regions, failing to include space-time interactions may lead to incorrect inferences about spatial effects or the effectiveness of policies. The inclusion of structured random effects ensures that residual spatial and temporal autocorrelation is accounted for, leading to more accurate estimates, reduced false positives, and more robust inference.

### 2.2. Why Bayesian?

Bayesian inference provides a coherent and flexible framework for modeling complex spatio-temporal processes. One of its key strengths is the ability to handle missing data in a principled manner [4,5]. Rather than relying on case-wise deletion or ad hoc imputation techniques, missing values are treated as additional parameters to be estimated within the model. This approach allows uncertainty arising from missingness to be incorporated into the posterior distribution, which leads to more credible and robust inference. This capability is especially important during public health emergencies such as the COVID-19 pandemic, when mobility and policy data are often incomplete, delayed, or inconsistently reported across regions and time.

Bayesian methods are also well suited to settings characterized by rapid or unprecedented changes. Because posterior updating integrates new observations with prior information, Bayesian models can capture sudden behavioral shifts or unexpected policy interventions without requiring full model re-estimation [6,7]. This adaptability is particularly valuable during events such as the COVID-19 pandemic, when historical patterns provide only partial guidance for contemporary dynamics. Incorporating prior information from recent local responses, such as behavioral trends from the previous day or policy actions in neighboring regions, helps stabilize inference when data are noisy, sparse, or only partially relevant to current conditions.

The R-INLA package offers an efficient platform for implementing complex Bayesian spatio-temporal models [11–13]. By using INLA, it provides fast and accurate estimation even in high-dimensional or irregular datasets. R-INLA supports a wide range of spatial structures, including Conditional Autoregressive (CAR) and Intrinsic CAR models. CAR priors borrow information from neighboring geographic units so that areas located close to one another are encouraged to have similar random effects, which captures spatial smoothing in an intuitive way. For temporal dynamics, the package accommodates components such as IID processes and autoregressive models including AR(1), where the value in one period depends on the previous period and therefore captures persistent temporal patterns. Most importantly, R-INLA enables the modeling of spatio-temporal interactions, allowing researchers to jointly estimate spatial dependence, temporal autocorrelation, and their interaction. This flexibility makes Bayesian spatio-temporal modeling particularly suitable for evaluating policy impacts that vary across both space and time.

### 2.3. Potential for policy evaluation

Recent studies have increasingly adopted Bayesian spatio-temporal modeling to address data sparsity, spatial correlation, and uncertainty in public health research. For example, Sianga et al. [14] used Bayesian generalized linear mixed models via INLA to analyze cardiovascular disease patterns in Tanzania, demonstrating the method’s strength in handling sparse spatial data. Similarly, Bauer et al. [15] employed Bayesian techniques to examine disparities in COVID-19 testing across fine geographic units, using spatial smoothing to leverage information from neighboring areas. Jeong et al. [16] applied a zero-inflated Poisson model to hepatitis A incidence in Korea, effectively modeling frequent zeros and spatio-temporal variation.

In parallel, other researchers have used Bayesian frameworks to manage model complexity and quantify uncertainty, especially when missing data are an issue. Zhang et al. [17,18] developed advanced models to estimate underreported COVID-19 deaths using Polya-Gamma augmentation for efficient inference and uncertainty propagation. Lemey et al. [19] show that Bayesian stochastic search variable selection can recover spatial transmission pathways in infectious disease dynamics, demonstrating how Bayesian spatiotemporal frameworks help model evolving spread in ways that relate directly to our analysis of COVID-19 mobility patterns. Lee et al. [20] use an INLA-based Bayesian spatiotemporal model to detect small-area risk heterogeneity, which illustrates how INLA enables joint spatial and temporal estimation similar to our approach. Amsalu et al. [21] apply hierarchical CAR models with space–time interaction terms to identify persistent tuberculosis hotspots, highlighting how Bayesian spatiotemporal methods capture evolving geographic risk in ways that parallel our goal of modeling spatial and temporal components of pandemic-related mobility.

Despite its growing adoption in epidemiology, Bayesian spatio-temporal modeling remains underutilized in policy evaluation research, even though it is particularly well-suited to capturing the complex dynamics of public interventions. Policies are often implemented in decentralized contexts and produce effects that vary not only across geographic regions [1] but also over time [2], and crucially, through their interaction. Accounting for these space-time interactions is essential to identifying localized and evolving policy impacts. Moreover, policy evaluations frequently rely on large administrative or behavioral datasets that are high-dimensional, unevenly distributed, and susceptible to missing data. Bayesian modeling frameworks offer a principled approach to these challenges by supporting flexible model structures, incorporating prior information, and treating missing values as estimable parameters within the model. This study shows the utility of Bayesian spatio-temporal modeling for health policy evaluation by applying it to assess the effects of stay-at-home orders on human mobility across the southern United States during the COVID-19 pandemic.

## 3. The impacts of social distancing policies on human mobilities

Social distancing, also referred to as physical distancing, is a central non-pharmaceutical intervention designed to limit disease transmission by reducing close interpersonal contact [22–24]. Common measures include work-from-home mandates, school closures, shelter-in-place orders, restrictions on large gatherings, and the closure of non-essential businesses [25–27]. Numerous studies have documented significant reductions in mobility following the implementation of these policies [28–31]. However, much of this literature treats policy impacts as static and homogeneous, often overlooking variation in effectiveness across geographic areas and over time.

Recent studies underscore the importance of accounting for both spatial and temporal dynamics. Elarde et al. [32], for example, identified distinct latent mobility patterns across U.S. counties during the COVID-19 pandemic, with geographically adjacent counties exhibiting similar trends, suggesting strong spatial dependence. These trends were also correlated with contextual factors such as political affiliation, population size, COVID-19 case and death rates, and unemployment levels. Likewise, Wei et al. [33] found that during the initial stay-at-home phase (late March–early May 2020), high-income groups reduced mobility more than low-income groups; after reopening, this pattern reversed. These findings indicate that policy effects are shaped not only by space or time individually, but also by their interaction, including how spatial patterns evolve over time and how temporal responses differ across regions.

Ignoring these space-time interactions risks yielding incomplete or biased estimates of policy effectiveness. To address this gap, the present study applies a Bayesian spatio-temporal modeling framework to evaluate the localized and dynamic effects of stay-at-home orders on human mobility across 1,422 counties in the southern United States. To our knowledge, this is the first study to formally estimate how the patterns of social distancing policies evolve as a function of space–time interaction, which is an essential but often overlooked dimension for understanding public health interventions in complex and heterogeneous settings.

## 4. Methods

### 4.1. Spatial and temporal scope

Our analysis focuses on 1,422 counties across Southern U.S. states, including Alabama, Arkansas, Delaware, the District of Columbia, Florida, Georgia, Kentucky, Louisiana, Mississippi, North Carolina, Oklahoma, South Carolina, Tennessee, Texas, Virginia, and West Virginia. This region was selected to balance computational feasibility with substantive relevance. The South exhibits considerable variation in demographics, healthcare access, and policy responses to COVID-19, making it a compelling setting for studying spatio-temporal dynamics. Additionally, limiting the geographic scope helps manage the computational demands of Bayesian spatio-temporal modeling, which become prohibitive at a national scale.

Our temporal scope spans February 2020 to June 2021, capturing the period when stay-at-home orders and social distancing policies were most actively implemented across the United States. This timeframe encompasses the initial emergence of COVID-19, the federal declaration of a public health emergency, and the peak of non-pharmaceutical interventions prior to the widespread availability of vaccines. Focusing on this period enables us to isolate the effects of social distancing measures on mobility at a time when such policies were most likely to influence public behavior.

### 4.2. Data

#### 4.2.1. Google mobility data.

This study uses county-level mobility data from Google’s Community Mobility Reports as the primary outcome variable. These high-resolution data are derived from anonymized smartphone location tracking and provide daily measurements of population movement across the globe. Mobility trends are reported relative to a baseline, defined as the median value for the corresponding day of the week during the 5-week period from January 3 to February 6, 2020. To avoid day-to-day volatility, particularly differences between weekdays and weekends, we follow Google’s recommendation to avoid direct comparisons across days and exclude weekends from our analysis [34]. This big data is aggregated to the monthly level to capture broader temporal trends, reduce short-term noise, and improve computational efficiency (S1 Table).

Among the six Google mobility categories—retail and recreation, groceries and pharmacies, parks, transit stations, workplaces, and residential—we focus on workplace and residential mobility, as these two provide relatively more stable and reliable measurements over time. Residential mobility captures the percentage change in time spent at home, while workplace mobility reflects changes in commuting patterns. If stay-at-home orders are effective, we would expect to observe a decrease in workplace mobility and a corresponding increase in residential mobility relative to the baseline period.

#### 4.2.2. Oxford COVID-19 Government Response Tracker.

This study also utilizes high-resolution policy data from the Oxford COVID-19 Government Response Tracker (OxCGRT) [35], a widely used big data source that documents daily government responses to the pandemic across countries and subnational jurisdictions. The dataset includes detailed records on the timing and scope of various non-pharmaceutical interventions, allowing for standardized comparison of policy implementation across regions. For our analysis, we focus on two types of stay-at-home policies: voluntary stay-at-home recommendations and legally enforced stay-at-home mandates.

In addition to these policies, we incorporate data on other relevant interventions from OxCGRT, including mask mandates, public information campaigns, and economic support measures. For stay-at-home orders, mask mandates, and public campaigns, we construct monthly policy intensity variables by counting the number of days each policy was active within a given month (S1 Table). For economic support, we use the monthly average of the OxCGRT Economic Support Index, a composite indicator (scaled 0–100) that captures the extent of household assistance through mechanisms such as direct cash transfers, wage subsidies, and debt relief [36].

#### 4.2.3. Control variables.

We include several covariates to account for demographic and socioeconomic factors that may influence mobility behavior and policy responsiveness (S1 Table). Data on COVID-19 cases were obtained from the Centers for Disease Control and Prevention (CDC), and vaccination rates were sourced from the OxCGRT. All other variables were drawn from the U.S. Census Bureau. Specifically, we control for population density, per capita GDP, average household size, unemployment rate, share of population over age 65, share of non-white population, and education level (measured as the number of people holding a bachelor’s degree or higher). These covariates help adjust for underlying structural and demographic differences across counties.

### 4.3. Methods

To estimate the effects of stay-at-home policies on human mobility, we specify four regression models with increasing levels of complexity. Each model progressively incorporates additional statistical structure to account for spatial dependence, temporal autocorrelation, and their interaction. All analyses are conducted at the county-month level, using a consistent set of policy, epidemiological, and demographic covariates across model specifications. The Bayesian models are estimated using the Integrated Nested Laplace Approximation (INLA) via the R-INLA package, which allows for efficient and accurate inference in latent Gaussian models without the computational intensity of traditional Markov Chain Monte Carlo (MCMC) methods.

#### 4.3.1. Baseline model (OLS Regression).

The baseline model is an OLS regression that estimates the association between stay-at-home policies and mobility without accounting for unobserved spatial or temporal correlations. The model is specified as:


$$\begin{array}{cc} {Mobility}_{cm}= & \;\beta_{\text{0}}+\beta_{\text{1}}{Stay}_{sm}^{\left(\text{Rec}\right)}+\;\beta_{\text{2}}{Stay}_{sm}^{\left(\text{Mand}\right)}+\;\beta_{\text{3}}{COVID}_{cm}+\;\beta_{\text{4}}{Vaccine}_{sm}+\beta_{Policy}^{\text{T}}X_{sm}^{\left(Policy\right)}\\ & +\beta_{Demo}^{\text{T}}X_{cy}^{\left(\text{Demo}\right)}+\varepsilon_{cm} \end{array}$$ (1)

where the dependent variable *Mobility*_*cm*_ refers to the percent change in either workplace or residential mobility in county *c* and month *m*. The key independent variables include the number of days of recommended (${Stay}_{sm}^{\left(\text{Rec}\right)}$) and mandatory (${Stay}_{sm}^{\left(\text{Mand}\right)}$) stay-at-home orders in the state s and month *m*, new COVID-19 cases per 100,000 people (*COVID*_*cm*_), and state-level vaccination rates (*Vaccine*_*sm*_). Additional covariates include other policy interventions ($X_{sm}^{\left(Policy\right)}$) and county-level demographics ($X_{cy}^{\left(\text{Demo}\right)}$). The error term $\varepsilon_{cm}$ is assumed to be independent and identically distributed (i.i.d.).

#### 4.3.2. Spatial model.

To address the violation of the independence assumption across space, the spatial model introduces two county-level random effects: a spatially structured effect *s*_*c*_, modeled with a Conditional Autoregressive (CAR) prior, and an unstructured random effect *µ*_*c*_, modeled as i.i.d. Gaussian noise:


$$\begin{array}{cc} {Mobility}_{cm}= & \;\beta_{\text{0}}+\beta_{\text{1}}{Stay}_{sm}^{\left(\text{Rec}\right)}+\;\beta_{\text{2}}{Stay}_{sm}^{\left(\text{Mand}\right)}+\;\beta_{\text{3}}{COVID}_{cm}+\;\beta_{\text{4}}{Vaccine}_{sm}+\beta_{Policy}^{\text{T}}X_{sm}^{\left(Policy\right)}\\ & +\beta_{Demo}^{\text{T}}X_{cy}^{\left(\text{Demo}\right)}+\varepsilon_{cm} \end{array}$$ (2)

The inclusion of spatial random effects controls for spatial autocorrelation driven by geographic clustering in both policy implementation and behavioral response. Failing to account for such autocorrelation can lead to spatial confounding, where spatially correlated omitted variables bias the estimated policy effects. The spatially structured effect *s*_*c*_ is modeled using a CAR prior, which encourages neighboring counties to have similar random effects and therefore provides intuitive spatial smoothing by borrowing information across adjacent areas. Specifically, we define neighborhood structure using a first-order rook contiguity matrix, where counties are considered neighbors if they share a common border. This specification allows information to be borrowed from adjacent areas, improving estimates in regions with sparse data and enabling localized smoothing. The unstructured effect *µ*_*c*_ captures residual heterogeneity at the county level that is not explained by spatial proximity and is assumed to follow an independent Gaussian distribution with constant variance. These components enable robust estimation of policy impacts while adjusting for unobserved spatial heterogeneity.

#### 4.3.3. Temporal model.

To address potential temporal dependence in mobility responses, we specify a temporal model that introduces a month-specific structured random effect *ηγ*_*m*_, modeled as a first-order autoregressive process AR(1). The model is given by:


$$\begin{array}{cc} {Mobility}_{cm}= & \;\beta_{\text{0}}+\beta_{\text{1}}{Stay}_{sm}^{\left(\text{Rec}\right)}+\;\beta_{\text{2}}{Stay}_{sm}^{\left(\text{Mand}\right)}+\;\beta_{\text{3}}{COVID}_{cm}+\;\beta_{\text{4}}{Vaccine}_{sm}\\ & +\beta_{Policy}^{\text{T}}X_{sm}^{\left(Policy\right)}+\beta_{Demo}^{\text{T}}X_{cy}^{\left(\text{Demo}\right)}+\gamma_{m}+\varepsilon_{cm} \end{array}$$ (3)

Here, *γ*_*m*_ captures unobserved temporal dynamics that evolve gradually over time. The AR(1) structure assumes that the effect in a given month depends partly on the previous month, making it well suited for capturing persistence in mobility behavior, such as gradual adaptation, fatigue, or risk normalization. This structure allows the model to account for autocorrelation in behavioral adaptation to the pandemic, such as lockdown fatigue or progressive risk normalization, which would be missed by models assuming temporal independence. We intentionally exclude fixed monthly effects and month-level i.i.d. random effects to avoid overparameterization and identifiability issues. Empirically, the AR(1) structure alone effectively captures the observed temporal dependence, with minimal differences in model fit statistics (DIC and WAIC) compared to more complex specifications. This modeling choice is also supported by theoretical expectations, as pandemic-related shifts in mobility are likely to follow autoregressive rather than abrupt or uncorrelated patterns within our study period (February 2020 to June 2021).

#### 4.3.4. Spatio-temporal model.

To rigorously evaluate the effects of stay-at-home policies during the COVID-19 pandemic, it is essential to model how policy responsiveness varies not only across space and time, but also through their interaction. The spatio-temporal model explicitly incorporates these complexities by introducing both structured random effects and flexible interaction terms. The full model is specified as:


$$\begin{array}{cc} {Mobility}_{cm}= & \;\beta_{\text{0}}+\beta_{\text{1}}{Stay}_{sm}^{\left(\text{Rec}\right)}+\;\beta_{\text{2}}{Stay}_{sm}^{\left(\text{Mand}\right)}+\;\beta_{\text{3}}{COVID}_{cm}+\;\beta_{\text{4}}{Vaccine}_{sm}+\beta_{Policy}^{\text{T}}X_{sm}^{\left(Policy\right)}\\ & +\beta_{Demo}^{\text{T}}X_{cy}^{\left(\text{Demo}\right)}+\sum_{m}\delta_{m}^{\text{T}}*(Z_{cm}+Z_{sm})+s_{c}+\mu_{c}+\gamma_{m}+\phi_{cm}+\varepsilon_{cm} \end{array}$$ (4)

This model captures three important dimensions of variation. First, the spatial random effects *s*_*c*_ and *µ*_*c*_ adjust for geographic clustering and unobserved county-level heterogeneity. Second, the temporal effect *γ*_*m*_ accounts for gradual changes in mobility over time. Third, the space–time interaction $\phi_{cm}$ allows each county to deviate from its long-run spatial pattern in ways that change across months.

The interaction term $\phi_{cm}$ plays an important role in spatio-temporal Bayesian models. It absorbs county-month–specific shocks that are not explained by the broader spatial or temporal structures. Because it is modeled as an i.i.d. Gaussian process, the term flexibly represents localized fluctuations while still allowing uncertainty to be appropriately propagated through the posterior distribution. This structure helps prevent short-term local changes, such as sudden behavioral shifts, localized outbreaks, or rapid policy adoption, from being incorrectly attributed to measured covariates.

Furthermore, we incorporate month-specific interaction terms $\sum_{m}\delta_{m}^{\text{T}}*(Z_{cm}+Z_{sm})$, allowing key policy and epidemiological covariates, such as stay-at-home orders, vaccination rates, mask mandates, and case rates, to exert time-varying effects. This enables the model to detect how the salience or effectiveness of these measures evolves over different pandemic phases. The inclusion of spatial random effects *s*_*c*_ and *µ*_*c*_, temporal autoregressive components *γ*_*m*_, and space-time interaction terms $\phi_{cm}$ ensures that the model captures the full complexity of policy diffusion and response across counties and over time. This specification aligns closely with the theoretical imperative to account for heterogeneous, evolving patterns of behavior under decentralized policy implementation.

## 5. Results

### 5.1. Descriptive results

Table 1 provides descriptive statistics for key variables included in the analysis, covering the period from February 2020 to June 2021. The dataset contains 22,254 observations for workplace mobility and 12,212 for residential mobility, indicating the number of county-month records with available Google mobility data. On average, workplace mobility decreased by 23.87% relative to the baseline (the median value from the five-week period of January 3 to February 6, 2020), while residential mobility showed an average increase of 7.15%, consistent with the expected effects of stay-at-home policies. The substantial presence of missing data points, particularly notable in residential mobility, highlights the strength of the Bayesian modeling approach, which adeptly handles incomplete data through spatio-temporal smoothing.

**Table 1:** Descriptive Statistics (Feb 2020 – Jun 2021).

	Obs.	Mean	Min	Max	S.D.
Workplace mobility	22,254	−23.87	−76.77	12.33	10.42
Residential mobility	12,212	7.15	−10.50	34.86	4.45
Stay-at-home (recommended)	24,157	20.15	0	31	13.68
Stay-at-home (mandatory)	24,157	4.53	0	31	9.59
Mask mandates	24,157	22.71	0	31	12.81
Public campaign	24,157	28.55	0	31	6.71
Economic support	24,157	0.35	−1.61	2.12	0.93
COVID-19 cases (cumulative)	23,494	4071	0	508464	16582.1
COVID-19 cases (new)	23,494	539.3	0	81642	2265.778
Vaccination rates	24,157	0.07	0.00	0.56	0.13
Population density	24,174	222.86	0.09	10415.07	704.59
Household size	24,174	2.57	1.89	4.84	0.23
Non-white population share	24,174	0.25	0.00	0.89	0.18
Unemployment rate	24,174	0.06	0.00	0.27	0.03
Share of population aged 65 and older	24,174	0.19	0.05	0.59	0.05
Share of population with a bachelor’s degree or higher	24,174	0.14	0.00	0.70	0.07

Note: This table presents descriptive statistics for all variables used in the analysis, covering 1,422 counties over 17 months (February 2020 to June 2021). Mobility measures are expressed as percentage changes relative to the Google-defined baseline period (January 3 to February 6, 2020). Stay-at-home order variables indicate the number of days per month each type of intervention was in effect. More detailed variable descriptions are reported in S1 Table.

Policy-related variables demonstrate substantial variability. Recommended stay-at-home orders were active for an average of 20.15 days per month (SD = 13.68), whereas mandatory stay-at-home orders had a lower monthly mean of 4.53 days and greater variability (SD = 9.59). Mask mandates averaged 22.71 active days per month, while public information campaigns had high consistency, averaging 28.55 active days monthly. The economic support index, capturing income support and debt relief measures, averaged 0.35 with a range from −1.61 to 2.12, reflecting significant variability across states. Epidemiological indicators reveal considerable variation across counties and over time. The dataset shows an average of 539.3 newly confirmed COVID-19 cases per 100,000 people per month, with a high degree of dispersion (SD = 2,265.78), reflecting the uneven geographic spread of the virus. The mean vaccination rate was relatively low at 7%, since the analysis period fell during the early phase of the pandemic.

**Fig 1:**
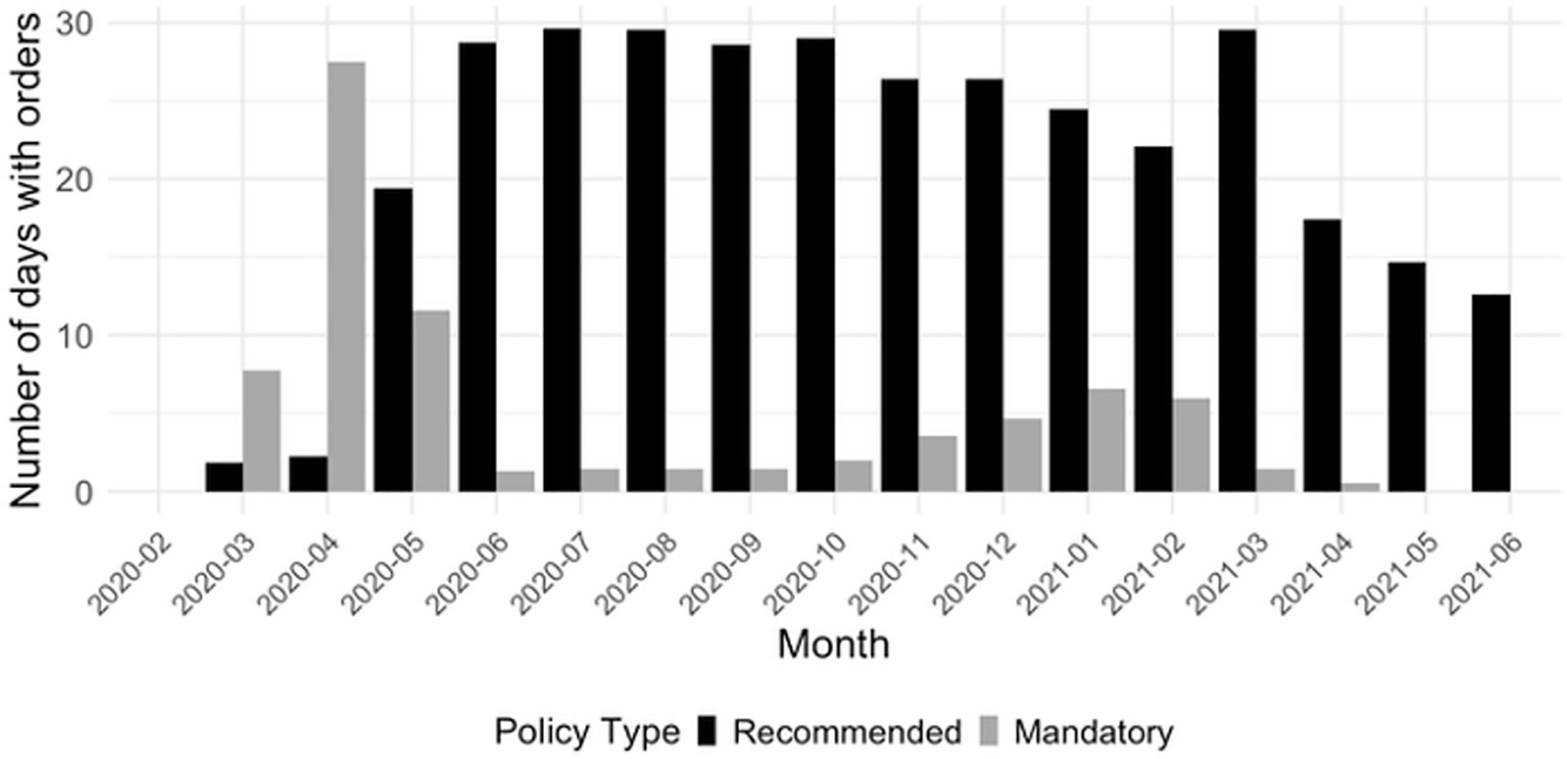
Monthly intensity of stay-at-home orders by policy type. Monthly number of days with recommended (black) and mandatory (dark grey) stay-at-home orders across southern U.S. states from February 2020 to June 2021.

Fig 1 displays the number of days each month in which stay-at-home orders were in effect across U.S. southern states, disaggregated by policy type: recommended (black) and mandatory (dark grey). At the onset of the pandemic in early 2020, states rapidly implemented both types of orders, with mandatory orders peaking in April and May 2020. Recommended orders also surged early but declined more sharply over time. Throughout 2020, mandatory stay-at-home policies remained prevalent, averaging over 20 days per month in most states until late fall. In contrast, recommended orders persisted at a lower and more variable rate. Both types of policies declined in frequency during 2021, reflecting policy relaxation and shifting public health strategies. This temporal variation in policy intensity provides critical context for interpreting behavioral changes in mobility.

**Fig 2:**
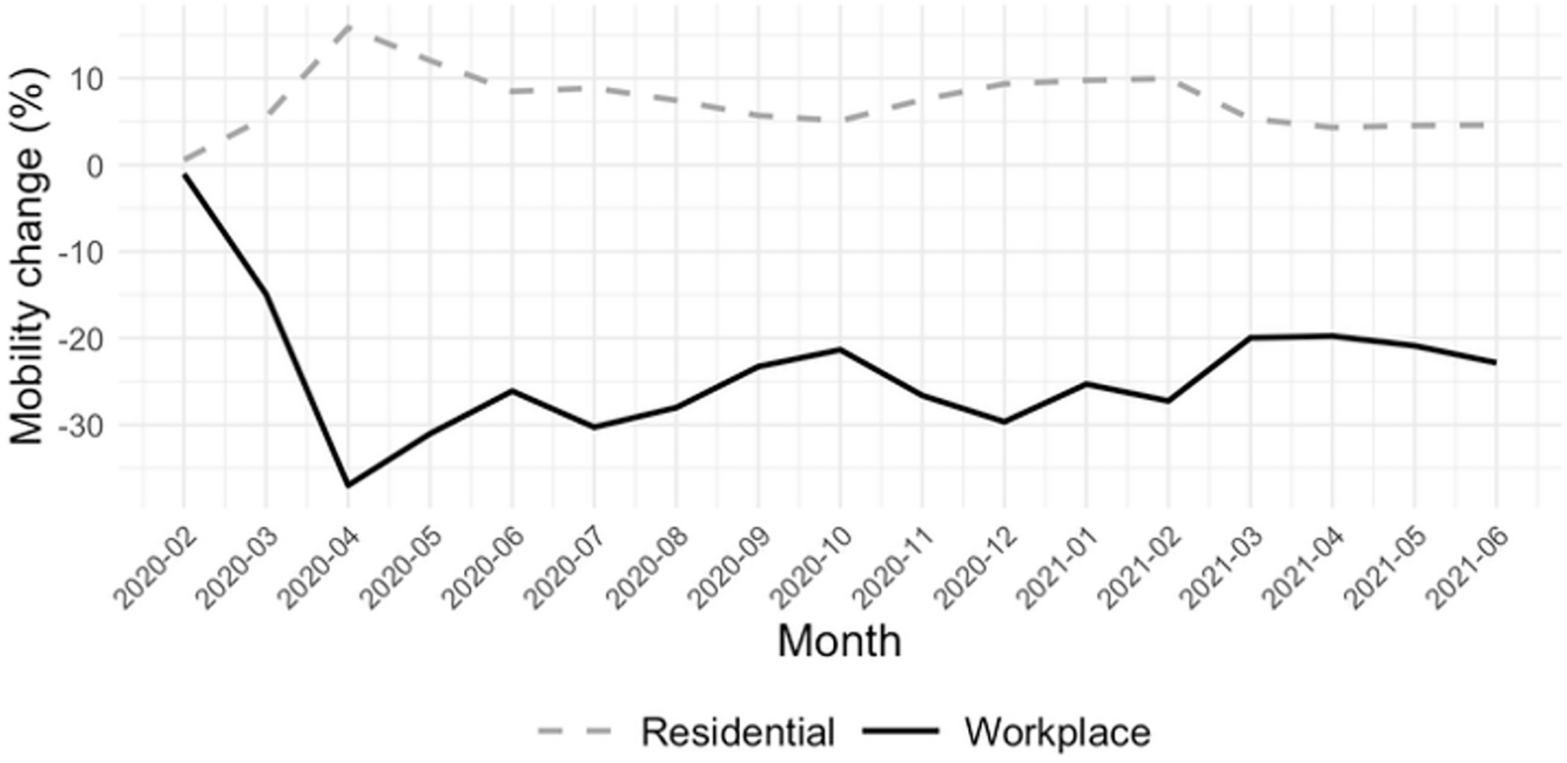
Monthly changes in residential and workplace mobility during the COVID-19 pandemic. Monthly average percent changes in mobility from February 2020 to June 2021 across southern U.S. counties, relative to the pre-pandemic baseline (January 3–February 6, 2020). Residential mobility is shown as a dashed gray line; workplace mobility as a solid black line.

Fig 2 presents the average monthly changes in mobility for two key domains, residential (red) and workplace (blue), relative to the pre-pandemic baseline (the median value from the 5‑week period Jan 3 – Feb 6, 2020). At the onset of the COVID-19 pandemic in March and April 2020, workplace mobility sharply declined by over 30%, while residential mobility simultaneously rose, reflecting widespread adherence to stay-at-home policies. Throughout 2020 and into 2021, residential mobility remained elevated compared to the baseline, indicating a sustained increase in time spent at home. In contrast, workplace mobility stayed well below baseline levels, with periodic fluctuations likely corresponding to waves of infections and subsequent policy tightening.

**Fig 3:**
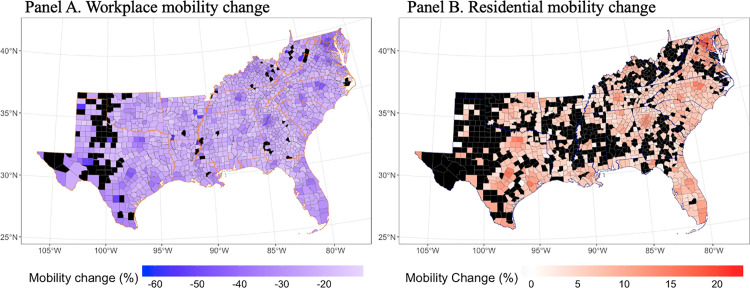
County-level mobility changes relative to pre-pandemic baseline. Panel A shows average workplace mobility changes by county, and Panel B presents residential mobility changes across 1,422 counties in the southern United States. Color gradients represent average percentage change from baseline (January 3–February 6, 2020), with darker blue indicating greater declines in workplace mobility and darker red indicating greater increases in residential mobility. Black-shaded counties indicate missing Google mobility data.

Building on these temporal trends, Fig 3 maps the geographic distribution of average mobility changes at the county level. Panel A depicts the average decline in workplace mobility across counties in the southern United States, with darker blue areas indicating more substantial reductions. Panel B shows corresponding changes in residential mobility, with warmer colors representing greater increases in time spent at home. Most counties experienced a decline in workplace mobility and a simultaneous increase in residential mobility during the early COVID-19 period (February 2020 – June 2021). The contrasting spatial distributions of workplace and residential mobility reveal uneven patterns of behavioral adaptation, shaped by local labor market structures, telework capacity, infrastructure, and demographic composition. Notably, several counties, particularly in Texas, Louisiana, and Georgia, are shaded in black, indicating missing data. These gaps highlight the utility of Bayesian hierarchical models, which can incorporate spatial smoothing to generate robust estimates in data-sparse regions. Taken together, these maps illustrate the spatial complexity of pandemic-era behavioral responses and reinforce the need for spatio-temporal modeling in evaluating policy effectiveness.

### 5.2. Baseline estimation results

Table 2 presents baseline OLS regression estimates of changes in workplace and residential mobility across U.S. counties from February 2020 to June 2021. Column (1) models percentage changes in workplace mobility, while Column (2) models changes in residential mobility, both relative to pre-pandemic baselines. Both recommended and mandatory stay-at-home orders were significantly associated with shifts in mobility behavior. Each additional day under a mandatory order corresponded to a 0.272 percentage point reduction in workplace mobility, compared to a 0.101 percentage point reduction under a recommended order. These findings indicate that stay-at-home policies effectively curtailed workplace visits and increased time spent at home, with mandatory orders exerting stronger behavioral constraints than voluntary recommendations.

**Table 2:** Baseline Estimation Results (OLS Regression).

	(1) Workplace Mobility	(2) Residential Mobility
Stay-at-home (recommended)	−0.101^***^ (0.006)	0.018^***^ (0.003)
Stay-at-home (mandatory)	−0.272^***^ (0.008)	0.114^***^ (0.004)
COVID-19 case (log)	−0.840^***^ (0.039)	0.433^***^ (0.021)
Vaccination rate	4.316^***^ (0.453)	−5.228^***^ (0.224)
Mask mandates	0.039^***^ (0.006)	−0.060^***^ (0.003)
Public campaign	−0.394^***^ (0.010)	0.074^***^ (0.006)
Economic support	−2.742^***^ (0.066)	1.554^***^ (0.035)
Population density (log)	0.718^***^ (0.064)	−0.003 (0.036)
Household size	−2.571^***^ (0.259)	2.088^***^ (0.139)
Non-white population share	1.233^***^ (0.278)	−0.406^***^ (0.174)
Unemployment rate	−24.298^***^ (2.127)	0.972 (1.594)
Share of population aged 65 and older	49.424^***^ (1.328)	−18.997^***^ (0.667)
Share of population with a bachelor’s degree or higher	−53.975^***^ (0.998)	22.844^***^ (0.493)
Intercept	−2.797^**^ (0.955)	−2.552^***^ (0.514)
Obs.	21,607	11,792
Adjusted R^2^	0.604	0.6841
F-statistic	2536^***^	1965^***^

Note: Standard errors are reported in parentheses.

*** p < 0.001, ** p < 0.01, * p < 0.05.

Epidemiological trends also showed strong associations with mobility outcomes. A one-unit increase in the natural logarithm of new monthly COVID-19 cases per 100,000 residents was associated with a 0.84 percentage point decrease in workplace mobility and a 0.433 percentage point increase in residential mobility. This suggests that individuals adapted their behavior in response to elevated perceived health risks by reducing out-of-home activities and spending more time at home. In contrast, a one percentage point increase in the vaccination rate was associated with a 4.32 percentage point increase in workplace mobility and a 5.23 percentage point decrease in residential mobility. These patterns indicate that broader vaccine coverage encouraged a return to pre-pandemic behaviors by boosting public confidence and lowering perceived vulnerability to infection.

Among other policy measures, mask mandates were linked to a 0.039 percentage point increase in workplace mobility and a 0.060 percentage point decrease in residential mobility per day. This may reflect individuals’ greater willingness to engage in out-of-home activities when protective measures are in place. Public information campaigns were associated with a 0.394 percentage point reduction in workplace mobility and a 0.074 percentage point increase in residential mobility per day, suggesting that consistent messaging promoted precautionary behavior and encouraged individuals to remain at home. Furthermore, each one-unit increase in the Economic Support Index, a composite measure of income support and debt relief, was associated with a 2.742 percentage point decline in workplace mobility and a 1.554 percentage point increase in residential mobility, implying that stronger financial support enabled greater compliance with stay-at-home recommendations by easing economic pressures.

While the models account for a substantial proportion of the variation in mobility outcomes, adjusted R^2^ values of 0.604 for workplace mobility and 0.684 for residential mobility, and all variance inflation factors (VIFs) are below the conventional threshold of 5 (S2 Table), several limitations remain. Most notably, the OLS framework does not account for potential spatial and temporal autocorrelation in the data. Since counties are geographically nested units and mobility patterns are likely to exhibit spillover effects or temporal dependencies, ignoring these structures may lead to biased coefficient estimates and underestimated standard errors. Diagnostics presented in S1 and S2 Figs confirm statistically significant spatial and temporal autocorrelation in the OLS residuals, reinforcing the need for more advanced modeling strategies. To address these concerns, subsequent sections employ Bayesian spatio-temporal models that explicitly incorporate spatial and temporal dependencies and allow for more robust inference in the presence of unobserved heterogeneity across both space and time.

### 5.4. Bayesian spatio-temporal modeling

Table 3 presents comparative results from four modeling strategies for estimating the effects of stay-at-home policies on workplace and residential mobilities across U.S. counties from February 2020 to June 2021. Column (1) reports estimates from a baseline OLS model that assumes independence across space and time. Column (2) introduces spatial dependence by incorporating a Bayesian spatial model with both structured (CAR) and unstructured (IID) county-level effects. Column (3) captures temporal autocorrelation through an autoregressive process of order 1 (AR(1)) across months. Column (4) combines both spatial and temporal dependencies within a spatio-temporal framework, allowing for complex space-time interactions.

**Table 3:** Estimation results.

	(1) Baseline Model (OLS)		(2) Spatial Model		(3) Temporal Model		(4) Spatio-Temporal Model	
	Workplace	Residential	Workplace	Residential	Workplace	Residential	Workplace	Residential
Stay-at-home (recommended)	−0.101^***^ (0.006)	0.018^***^ (0.003)	−0.072^†^ [-0.082; -0.061]	0.002 [-0.004; 0.008]	−0.013^†^ [-0.026; -0.001]	0.007^†^ [0.002; 0.013]	−0.171 [-6.104; 5.761]	0.051 [-5.882; 5.983]
Stay-at-home (mandatory)	−0.272^***^ (0.008)	0.114^***^ (0.004)	−0.284^†^ [-0.297; -0.270]	0.126^†^ [0.118; 0.133]	−0.052^†^ [-0.068; -0.036]	0.012^†^ [0.005; 0.019]	−0.253 [-6.718; 6.212]	0.077 [-6.388; 6.541]
COVID-19 (log)	−0.840^***^ (0.039)	0.433^***^ (0.021)	−0.958^†^ [-1.020; -0.895]	0.455^†^ [0.420; 0.490]	−0.018 [-0.067; 0.031]	0.149^†^ [0.126; 0.171]	−0.314 [-5.070; 4.442]	0.190 [-4.566; 4.946]
Other policies	Yes	Yes	Yes	Yes	Yes	Yes	Yes	Yes
Other covariates	Yes	Yes	Yes	Yes	Yes	Yes	Yes	Yes
Gaussian Precision			0.039^†^ [0.039; 0.04]	0.225^†^ [0.219; 0.231]	0.034^†^ [0.034; 0.035]	0.277^†^ [0.270; 0.288]	0.386^†^ [0.092; 1.158]	1.12^†^ [0.240; 3.20]
County IID			0.042^†^ [0.033; 0.049]	0.491^†^ [0.436; 0.551]			0.046^†^ [0.042; 0.050]	0.557^†^ [0.373; 0.720]
CAR (IID)			12.8^†^ [0.969; 74.4]	4354.859^†^ [10.53; 30500]			3278.69^†^ [687.2; 9975.2]	92.9^†^ [1.162; 625.0]
CAR (Spatial)			38100000^†^ [40.2; 24900000]	1934.104^†^ [71.122; 6620]			994.442^†^ [106.3; 4115.4]	181,000^†^ [78.0; 939,000]
Month AR (1)					0.033^†^ [0.021; 0.048]	0.146^†^ [0.110; 0.227]	38.536^†^ [4.60; 137.69]	1,140^†^ [0.716; 7,370]
Month AR (1) ρ					0.385^†^ [0.127; 0.596]	0.445^†^ [0.107; 0.681]	0.8^†^ [0.44; 0.97]	0.261 [-0.948; 0.992]
Space Time IID							0.113^†^ [0.059; 0.189]	2.02^†^ [0.304; 7.45]
Adjusted R^2^	0.604	0.6841						
F-statistic	2536^***^	1965^***^						
DIC			136577.54	53558.22	138252.62	50442.88	108530.54	40032.33
WAIC			136628.84	53576.20	138244.02	50445.92	106465.55	41210.03

Note: In Column (1), standard errors are reported in parentheses (^***^p < 0.001, ^**^p < 0.01, ^*^p < 0.05), and the estimates correspond to the OLS specification in Equation (1). Columns (2)–(4) report posterior means from the Bayesian models in Equations (2)–(4), with 95% Bayesian credible intervals shown in brackets. Posterior means marked with ^†^ indicate that the 95% credible interval does not include zero, signifying statistical significance. “Other policies” include mask mandates, public campaigns, and the economic support index. “Other covariates” include county-level controls: vaccination rate, population density (log), household size, non-white share, unemployment rate, population over age 65, and population with a bachelor’s degree or higher. Full results are provided in S5–S7 Tables.

While the coefficients in Column (1) represent point estimates obtained through frequentist OLS regression, the posterior means in Columns (2) through (4) reflect Bayesian estimates—that is, the expected values of the parameters given both the prior distribution and the observed data. These posterior means are derived from the posterior distribution, which combines prior beliefs with the likelihood of the data using Bayes’ theorem. As such, they represent a probabilistically weighted average of all plausible parameter values. Unlike OLS estimates, which are accompanied by p-values for significance testing, Bayesian estimates are evaluated through 95% credible intervals, which indicate the range within which the parameter lies with a specified probability, given the data and model assumptions.

This difference arises from the distinct ways each framework conceptualizes the parameter β. In the frequentist OLS framework, β is treated as a fixed but unknown quantity, and coefficients are interpreted as point estimates. For example, “a one-unit increase in X is associated with an estimated β-unit change in Y.” Uncertainty is assessed through p-values and confidence intervals, which describe how the estimator would vary across repeated samples. In contrast, the Bayesian framework treats β as a random variable with a posterior distribution, and interpretation is explicitly probabilistic. For example, “given the data and prior assumptions, β is most likely to lie near its posterior mean, with a 95 percent probability that it falls within the corresponding credible interval.” In this approach, credible intervals quantify uncertainty directly about the parameter itself.

In addition to differences in interpretation, the two frameworks differ in how they evaluate model performance. The explanatory power of the OLS model is supported by significant F-statistics and relatively high adjusted R-squared values. In contrast, the Bayesian models do not rely on frequentist significance tests but instead assess model performance and parameter certainty through posterior distributions. One key diagnostic is the Gaussian precision parameter, which reflects the inverse of the residual variance. In all Bayesian models, the Gaussian precision estimates have narrow 95% credible intervals that exclude zero, indicating that the models provide stable and precise estimates of the residual variation and are well-calibrated to the observed data.

Substantively, across the OLS (Column 1), spatial (Column 2), and temporal (Column 3) models, both recommended and mandatory stay-at-home orders (measured in days per month) and logged COVID-19 case counts (per 100,000 residents) are generally associated with reduced workplace mobility and increased residential mobility. One exception is the non-significant effect of recommended orders on residential mobility in the spatial model. The estimated magnitudes vary across models: for workplace mobility, the effect of recommended orders declines from −0.101 in the OLS model to −0.072 in the spatial model and −0.013 in the temporal model. For mandatory orders, the estimates range from −0.272 (OLS) to −0.284 (spatial) and −0.052 (temporal). These differences suggest that accounting for spatial and temporal dependencies attenuates estimated effect sizes, likely by addressing unobserved heterogeneity and serial correlation that bias OLS estimates.

In the fully specified spatio-temporal model (Column 4), the estimated effects of stay-at-home policies and COVID-19 case rates are no longer statistically distinguishable from zero, as all 95% credible intervals cross zero. This attenuation indicates that once spatial and temporal autocorrelations, as well as their interactions, are explicitly modeled, the apparent direct associations are absorbed by latent spatio-temporal processes. Specifically, the random effects components (County IID, CAR, AR(1), and Space-Time IID) in the spatio-temporal model further illuminate these dynamics. Moreover, even in a sensitivity analysis in which we re-estimated the models after excluding counties with more than 30% missing observations (S8 Table), the policy coefficients remained statistically insignificant. This confirms that the loss of statistical significance stems from the proper modeling of spatial and temporal dependence rather than from missing-data artifacts. Accordingly, the conclusions are robust to missing-data restrictions, and missingness does not materially affect the estimated policy effects.

First, the County IID term captures idiosyncratic, unstructured variation across counties that is not spatially correlated. Its credible interval excludes zero, confirming substantial unexplained county-level heterogeneity. Second, the CAR (structured spatial effect) models the influence of neighboring counties, with strong precision values indicating significant spatial clustering, suggestive of regional policy diffusion, shared infrastructure, or cultural proximity. Third, the Month AR(1) term captures time series dependence, with a significant autocorrelation parameter (ρ), reflecting behavioral inertia or policy lags across months. Lastly, the Space-Time IID term accounts for interaction effects unique to specific county-month combinations, such as localized outbreaks or targeted public health responses, that are not explained by purely spatial or temporal patterns.

Model comparison metrics further underscore the importance of accounting for spatio-temporal dependencies. Both the Deviance Information Criterion (DIC) and the Watanabe-Akaike Information Criterion (WAIC) evaluate model performance by balancing goodness of fit with model complexity, where lower values indicate superior model performance. Among the four specifications, the spatio-temporal model yields the lowest DIC (108,530.54 for workplace mobility; 40,032.33 for residential mobility) and WAIC (106,465.55; 41,210.03), suggesting it provides the best overall fit while avoiding overfitting. These improvements relative to the spatial and temporal models highlight the analytical value of jointly modeling space-time interactions to capture latent structures that influence mobility behavior.

### 5.5. Predicted changes in mobility from the Bayesian spatio-temporal model

**Fig 4:**
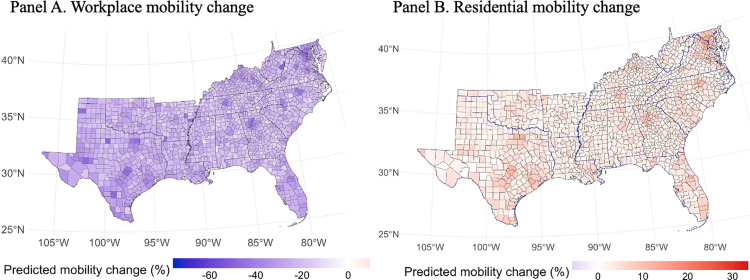
Bayesian-predicted county-level mobility changes from the spatio-temporal model. Panel A displays predicted workplace mobility, and Panel B shows predicted residential mobility derived from the Bayesian spatio-temporal model specified in Equation (4). Darker purple shades represent larger workplace mobility declines, and deeper red tones represent larger increases in residential mobility. The model incorporates CAR spatial structure, AR(1) temporal dependence, and a space–time interaction term, producing smoothed predictions that account for spatial and temporal dependence while accommodating missing data.

Fig 4 illustrates predicted county-level changes in mobility behavior derived from the Bayesian spatio-temporal model, disaggregated by mobility type. Panel A presents predicted changes in workplace mobility, with darker shades of purple indicating greater reductions relative to pre-pandemic baselines. Panel B shows predicted changes in residential mobility, where deeper red hues reflect increased time spent at home. The model captures considerable spatial heterogeneity in mobility responses, with particularly pronounced shifts observed in the eastern counties.

In contrast to the observed mobility data depicted in Fig 3, which contains substantial missingness in many counties, the Bayesian predictions in Fig 4 offer a smoother and more spatially comprehensive depiction of mobility trends. This advantage stems from one of the core strengths of Bayesian spatio-temporal modeling: its ability to handle missing data through formal probabilistic inference. Rather than resorting to listwise deletion or ad hoc imputation, the model treats missing values as latent variables and estimates them as part of the posterior distribution [4,5]. This approach not only retains all available information but also quantifies uncertainty due to missingness, enhancing the robustness and credibility of the estimates.

Such a capability is especially critical during public health emergencies like the COVID-19 pandemic, where mobility and policy data are often incomplete or inconsistently reported across space and time. By leveraging spatial and temporal correlations, the Bayesian spatio-temporal framework enables more reliable estimation and inference, thereby offering valuable insight into the population’s behavioral responses even in data-sparse settings.

## 6. Discussion

Our findings demonstrate that accounting for spatial, temporal, and especially space-time interaction effects can substantially alter conclusions about the effectiveness of stay-at-home orders. In simpler models, such as OLS or those capturing only spatial or temporal dependencies, policy effects appeared statistically significant and aligned with conventional narratives suggesting that stringent policies reduced mobility. However, once latent spatio-temporal structures were incorporated, many of these associations became statistically indistinguishable from zero. This attenuation suggests that some previously observed policy effects may reflect underlying regional trends or correlated local behaviors, rather than direct causal impacts of the policies themselves. Neglecting the interactive nature of space and time can therefore lead to biased or misleading conclusions about the true impact of public interventions.

Additionally, this study highlights the strengths of the Bayesian framework in dealing with common data challenges in real-world policy evaluation. Data sparsity and missingness, particularly common in county-level mobility data during the early pandemic, pose significant barriers to inference. By treating missing mobility values as parameters to be estimated rather than omitted or imputed externally, our Bayesian model formally integrates the uncertainty associated with data incompleteness into its inference process. This approach yields a more robust and spatially continuous estimate of mobility trends, particularly in counties with incomplete observations, as visually demonstrated by the contrast between Figs 3 and 4.

This study contributes to the existing literature in two important ways. First, while Bayesian spatio-temporal models have been widely applied in epidemiological and infectious disease research [14 –19], their use in public policy evaluation remains limited. By applying this framework to the analysis of non-pharmaceutical interventions during the COVID-19 pandemic, we extend its applicability to evaluating policy interventions that unfold unevenly across space and time. Second, this study contributes to a growing body of research on the effects of social distancing policies on population mobility. While prior studies [28–31] have reported statistically significant reductions in mobility following the implementation of stay-at-home orders, our findings suggest that such conclusions may be sensitive to modeling choices, particularly the extent to which spatial and temporal dependencies are taken into account.

Before concluding, we should acknowledge several limitations. First, our geographic focus on counties in the southern United States may limit the generalizability of our findings to regions with different demographic compositions, public health infrastructures, or governance structures. Second, although Google’s mobility data provide valuable insights into aggregate movement trends, they do not capture individual-level behaviors or the specific motivations behind compliance with policy directives. Third, despite the Bayesian framework’s capacity to manage missing data rigorously, the quality of inference may still be influenced by the degree and geographic distribution of data gaps. Fourth, the computational demands of fitting high-dimensional spatio-temporal INLA models constrained our ability to implement certain robustness checks, such as weekly aggregation. Weekly models exceeded the available computational memory due to the large spatial adjacency matrices and space–time random-effects structures required, and thus could not be reliably estimated. Finally, while our models adjust for observed covariates and incorporate unobserved heterogeneity through random effects, they do not establish causal relationships in a quasi-experimental or counterfactual sense. Future research would benefit from integrating spatio-temporal models with causal inference strategies such as difference-in-differences designs, instrumental variables, or synthetic control methods to improve the identification of policy effects.

## Supporting information

S1 Fig Moran’s I scatterplots for residuals from the baseline OLS models. Panel A presents the Moran’s I scatterplot for residuals from the baseline OLS model of workplace mobility, while Panel B shows the corresponding scatterplot for residential mobility. In both panels, the upward-sloping trend lines indicate positive spatial autocorrelation, suggesting that counties with higher-than-expected mobility tend to be surrounded by similarly high-mobility neighbors—and vice versa for lower values. The observed Moran’s I statistics are 0.163 for workplace mobility and 0.185 for residential mobility, both statistically significant at the 99% confidence level (Appendix Table 3).
(DOCX)

S2 Fig Autocorrelation Function (ACF) plots for OLS residuals. Panel A presents the ACF plot for residuals from the baseline OLS model of workplace mobility, while Panel B shows the corresponding plot for residential mobility. In both panels, multiple lagged autocorrelations exceed the 95% confidence bounds (dashed lines), indicating statistically significant serial correlation. The slow decay pattern suggests persistent temporal dependence, violating the assumption of uncorrelated residuals. These results are corroborated by formal diagnostics—including the Breusch-Godfrey and Durbin-Watson tests—which confirm significant positive autocorrelation at the 99% confidence level (Appendix Table 4).
(DOCX)

S1 Table Variable description. Note: This table provides definitions, units of analysis, and data sources for all variables used in the Bayesian spatio-temporal models, including mobility outcomes, COVID-19 indicators, policy measures, and demographic and socioeconomic covariates. Data (Sources: ^i^ Google Mobility Reports, ^ii^ CDC, ^iii^ Oxford COVID-19 Government Response Tracker, ^iv^ U.S. Census Bureau).
(DOCX)

S2 Table Variance Inflation Factors (VIFs) from OLS regression models. Note: This table reports the variance inflation factors for all covariates included in the workplace and residential mobility OLS regression models, used to assess potential multicollinearity among predictors.
(DOCX)

S3 Table Spatial autocorrelation diagnostics. Note: This table presents Moran’s I statistics and residual dispersion measures for both the OLS and Bayesian spatio-temporal models, assessing the presence of spatial autocorrelation in workplace and residential mobility outcomes. ^***^p < 0.001, ^**^p < 0.01, ^*^p < 0.05.
(DOCX)

S4 Table Temporal autocorrelation diagnostics. Note: This table reports the Breusch–Godfrey test statistics and Durbin–Watson values for the workplace and residential mobility OLS models, evaluating the presence of temporal autocorrelation in the residuals. ^***^p < 0.001, ^**^p < 0.01, ^*^p < 0.05.
(DOCX)

S5 Table Full results from Bayesian spatial model. Note: In Column (1), standard errors are reported in parentheses (^***^p < 0.001, ^**^p < 0.01, ^*^p < 0.05). Columns (2) – (4) present the posterior means of the estimated coefficients, with 95% Bayesian credible intervals shown in brackets. Posterior means marked with † indicate that the 95% credible interval does not include zero, signifying statistical significance.
(DOCX)

S6 Table Full results from Bayesian temporal model. Note: In Column (1), standard errors are reported in parentheses (^***^p < 0.001, ^**^p < 0.01, ^*^p < 0.05). Columns (2) – (4) present the posterior means of the estimated coefficients, with 95% Bayesian credible intervals shown in brackets. Posterior means marked with † indicate that the 95% credible interval does not include zero, signifying statistical significance.
(DOCX)

S7 Table Full results from Bayesian spatio-temporal model. Note: In Column (1), standard errors are reported in parentheses (^***^p < 0.001, ^**^p < 0.01, ^*^p < 0.05). Columns (2) – (4) present the posterior means of the estimated coefficients, with 95% Bayesian credible intervals shown in brackets. Posterior means marked with † indicate that the 95% credible interval does not include zero, signifying statistical significance.
(DOCX)

S8 Table Bayesian spatio-temporal model estimates after excluding counties with more than 30% missing mobility data. Note: As a sensitivity test, the models were re-estimated after excluding counties with more than 30% missing observations. In Column (1), standard errors are reported in parentheses (^***^p < 0.001, ^**^p < 0.01, ^*^p < 0.05). Columns (2) – (4) present the posterior means of the estimated coefficients, with 95% Bayesian credible intervals shown in brackets. Posterior means marked with † indicate that the 95% credible interval does not include zero, signifying statistical significance.
(DOCX)
