# Supplementary material for: Bayesian spatio-temporal modeling for policy evaluation: Sensitivity of policy effect estimates in the context of COVID-19 stay-at-home orders
Source: PLoS One. 2026 Feb 10;21(2):e0339196. doi: 10.1371/journal.pone.0339196 (PMC12890128; doi:10.1371/journal.pone.0339196)
Supplement: S1 Fig — Panel A presents the Moran’s I scatterplot for residuals from the baseline OLS model of workplace mobility, while Panel B shows the corresponding scatterplot for residential mobility. In both panels, the upward-sloping trend lines indicate positive spatial autocorrelation, suggesting that counties with higher-than-expected mobility tend to be surrounded by similarly high-mobility neighbors—and vice versa for lower values. The observed Moran’s I statistics are 0.163 for workplace mobility and 0.185 for residential mobility, both statistically significant at the 99% confidence level (Appendix Table 3). (DOCX) [file pone.0339196.s001.docx]

**Supporting Information**

| Panel A. Workplace mobility residuals  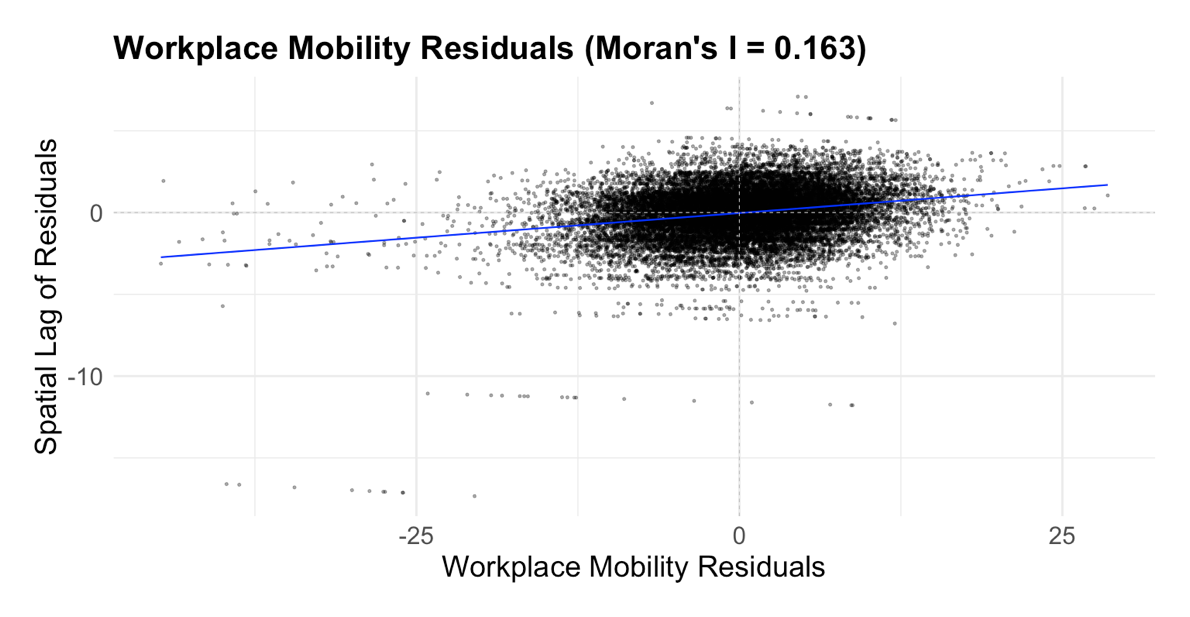 |
| --- |
| Panel B. Residential mobility residuals  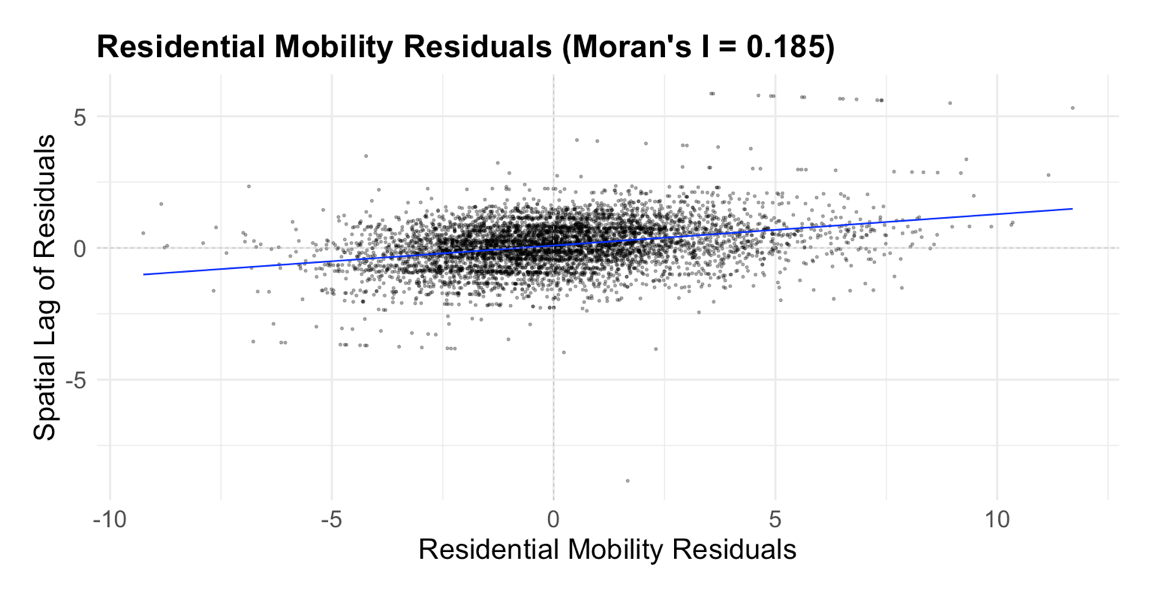 |

**S1 Fig. Moran’s I Scatterplots for Residuals from the Baseline OLS Models**

Panel A presents the Moran’s I scatterplot for residuals from the baseline OLS model of workplace mobility, while Panel B shows the corresponding scatterplot for residential mobility. In both panels, the upward-sloping trend lines indicate positive spatial autocorrelation, suggesting that counties with higher-than-expected mobility tend to be surrounded by similarly high-mobility neighbors—and vice versa for lower values. The observed Moran’s I statistics are 0.163 for workplace mobility and 0.185 for residential mobility, both statistically significant at the 99% confidence level (Appendix Table 3).
