# Supplementary material for: Bayesian spatio-temporal modeling for policy evaluation: Sensitivity of policy effect estimates in the context of COVID-19 stay-at-home orders
Source: PLoS One. 2026 Feb 10;21(2):e0339196. doi: 10.1371/journal.pone.0339196 (PMC12890128; doi:10.1371/journal.pone.0339196)
Supplement: S2 Fig — Panel A presents the ACF plot for residuals from the baseline OLS model of workplace mobility, while Panel B shows the corresponding plot for residential mobility. In both panels, multiple lagged autocorrelations exceed the 95% confidence bounds (dashed lines), indicating statistically significant serial correlation. The slow decay pattern suggests persistent temporal dependence, violating the assumption of uncorrelated residuals. These results are corroborated by formal diagnostics—including the Breusch-Godfrey and Durbin-Watson tests—which confirm significant positive autocorrelation at the 99% confidence level (Appendix Table 4). (DOCX) [file pone.0339196.s002.docx]

**Supporting Information**


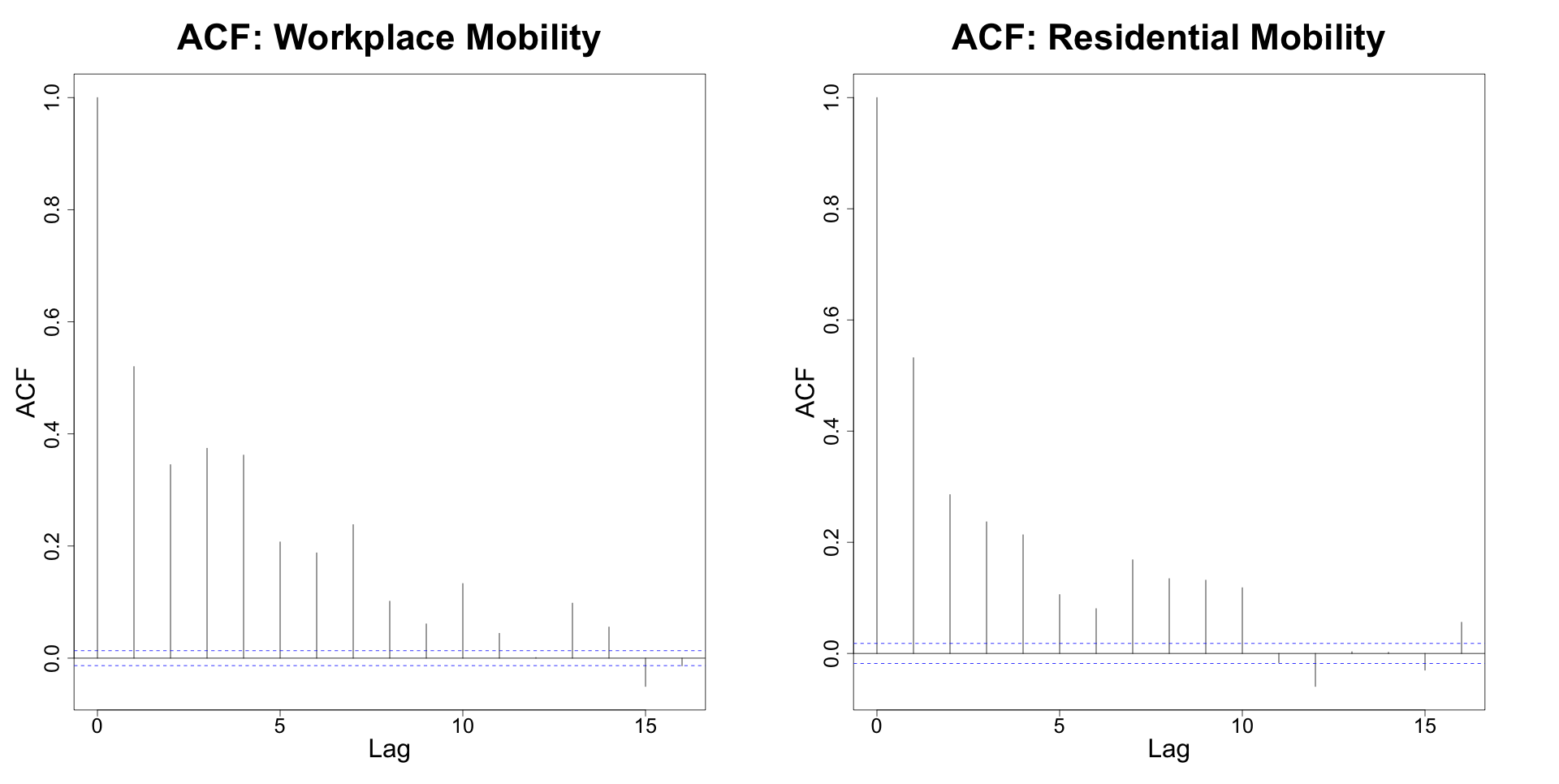


**S2 Fig. Autocorrelation Function (ACF) Plots for OLS Residuals**

Panel A presents the ACF plot for residuals from the baseline OLS model of workplace mobility, while Panel B shows the corresponding plot for residential mobility. In both panels, multiple lagged autocorrelations exceed the 95% confidence bounds (dashed lines), indicating statistically significant serial correlation. The slow decay pattern suggests persistent temporal dependence, violating the assumption of uncorrelated residuals. These results are corroborated by formal diagnostics—including the Breusch-Godfrey and Durbin-Watson tests—which confirm significant positive autocorrelation at the 99% confidence level (Appendix Table 4).
