# Supplementary material for: Bayesian spatio-temporal modeling for policy evaluation: Sensitivity of policy effect estimates in the context of COVID-19 stay-at-home orders
Source: PLoS One. 2026 Feb 10;21(2):e0339196. doi: 10.1371/journal.pone.0339196 (PMC12890128; doi:10.1371/journal.pone.0339196)
Supplement: S1 Table — Note: This table provides definitions, units of analysis, and data sources for all variables used in the Bayesian spatio-temporal models, including mobility outcomes, COVID-19 indicators, policy measures, and demographic and socioeconomic covariates. Data (Sources: i Google Mobility Reports, ii CDC, iii Oxford COVID-19 Government Response Tracker, iv U.S. Census Bureau). (DOCX) [file pone.0339196.s003.docx]

**Supporting Information**

**S1 Table. Variable Description**

| **Variables** | **Description** | **Unit of Analysis** |
| --- | --- | --- |
| Workplace mobility | Percent change in visits to workplaces from baseline ^i^ | County-Month |
| Residential mobility | Percent change in time spent at residences from baseline ^i^ | County-Month |
| COVID-19 cases | Number of newly confirmed COVID-19 cases per 100,000^ii^ | County-Month |
| Vaccination rate | Proportion of the population reported as vaccinated against COVID-19^iii^ | State-Month |
| Stay-at-home order (recommended) | Number of days with recommended stay-at-home orders ^iii^ | State-Month |
| Stay-at-home order (mandatory) | Number of days with mandatory stay-at-home orders ^iii^ | State-Month |
| Mask mandates | Number of days with face covering mandates ^iii^ | State-Month |
| Public campaign | Number of days public information campaigns were active ^iii^ | State-Month |
| Economic Support Index | Average index measuring income support and debt/contract relief provided to households during the pandemic ^iii^ | State-Month |
| Population density | Population per square mile ^iv^ | County-Year |
| Per capita GDP | Per capita GDP in U.S. dollars ^iv^ | County-Year |
| Household size | Average number of individuals per household ^iv^ | County-Year |
| Non-white share | Percentage of the population identifying as non-white ^iv^ | County-Year |
| Unemployment rate | Percentage of the labor force that is unemployed ^iv^ | County-Year |
| Population over age 65 | Percentage of the population aged 65 and older ^iv^ | County-Year |
| Education level | Number of people who hold a bachelor's degree or higher ^iv^ | County-Year |
| Note: This table provides definitions, units of analysis, and data sources for all variables used in the Bayesian spatio-temporal models, including mobility outcomes, COVID-19 indicators, policy measures, and demographic and socioeconomic covariates. Data (Sources: ^i^ Google Mobility Reports, ^ii^ CDC, ^iii^ Oxford COVID-19 Government Response Tracker, ^iv^ U.S. Census Bureau) | | |
