# Supplementary material for: Bayesian spatio-temporal modeling for policy evaluation: Sensitivity of policy effect estimates in the context of COVID-19 stay-at-home orders
Source: PLoS One. 2026 Feb 10;21(2):e0339196. doi: 10.1371/journal.pone.0339196 (PMC12890128; doi:10.1371/journal.pone.0339196)
Supplement: S2 Table — Note: This table reports the variance inflation factors for all covariates included in the workplace and residential mobility OLS regression models, used to assess potential multicollinearity among predictors. (DOCX) [file pone.0339196.s004.docx]

**Supporting Information**

**S2 Table. Variance Inflation Factors (VIFs) from OLS Regression Models**

| Variable | (1) Workplace Mobility | (2) Residential Mobility |
| --- | --- | --- |
| Stay-at-home (recommendation) | 3.98 | 3.85 |
| Stay-at-home (mandatory) | 3.21 | 3.29 |
| COVID-19 case (log) | 3.76 | 3.90 |
| Vaccination | 1.85 | 1.82 |
| Mask mandates | 2.91 | 2.89 |
| Public campaign | 2.35 | 2.50 |
| Economic support | 1.98 | 1.89 |
| Population density (log) | 2.87 | 2.71 |
| Household size | 1.56 | 1.54 |
| Non-white share | 1.23 | 1.41 |
| Unemployment rate | 1.27 | 1.40 |
| Population over age 65 | 1.88 | 1.90 |
| Education level | 2.05 | 2.14 |
| Note: This table reports the variance inflation factors for all covariates included in the workplace and residential mobility OLS regression models, used to assess potential multicollinearity among predictors. | | |
