# Supplementary material for: Bayesian spatio-temporal modeling for policy evaluation: Sensitivity of policy effect estimates in the context of COVID-19 stay-at-home orders
Source: PLoS One. 2026 Feb 10;21(2):e0339196. doi: 10.1371/journal.pone.0339196 (PMC12890128; doi:10.1371/journal.pone.0339196)
Supplement: S3 Table — Note: This table presents Moran’s I statistics and residual dispersion measures for both the OLS and Bayesian spatio-temporal models, assessing the presence of spatial autocorrelation in workplace and residential mobility outcomes. ***p < 0.001, **p < 0.01, *p < 0.05. (DOCX) [file pone.0339196.s005.docx]

**Supporting Information**

**S3 Table. Spatial Autocorrelation Diagnostics**

|  | OLS Model | | Spatio-Temporal Model | |
| --- | --- | --- | --- | --- |
|  | (1)  Workplace Mobility | (2)  Residential Mobility | (3)  Workplace Mobility | (4)  Residential Mobility |
| Observed Moran’s I | 0.163^***^ | 0.185^***^ | -0.010 | -0.015 |
| Residual Minimum | -46.28 | -17.05 | -21.22 | -5.65 |
| Residual Maximum | 28.51 | 11.70 | 22.32 | 2.56 |
| Range | 74.79 | 28.76 | 43.54 | 8.21 |
| Note: This table presents Moran’s I statistics and residual dispersion measures for both the OLS and Bayesian spatio-temporal models, assessing the presence of spatial autocorrelation in workplace and residential mobility outcomes. ^***^p < 0.001, ^**^p < 0.01, ^*^p < 0.05 | | | | |
