# Supplementary material for: Bayesian spatio-temporal modeling for policy evaluation: Sensitivity of policy effect estimates in the context of COVID-19 stay-at-home orders
Source: PLoS One. 2026 Feb 10;21(2):e0339196. doi: 10.1371/journal.pone.0339196 (PMC12890128; doi:10.1371/journal.pone.0339196)
Supplement: S4 Table — Note: This table reports the Breusch–Godfrey test statistics and Durbin–Watson values for the workplace and residential mobility OLS models, evaluating the presence of temporal autocorrelation in the residuals. ***p < 0.001, **p < 0.01, *p < 0.05. (DOCX) [file pone.0339196.s006.docx]

**Supporting Information**

**S4 Table. Temporal Autocorrelation Diagnostics**

|  | (1) Workplace Mobility | (2) Residential Mobility |
| --- | --- | --- |
| Breusch-Godfrey Test (df=1) | 6214.2^***^ | 3509.2^***^ |
| Durbin-Watson Statistics | 0.9597^***^ | 0.9360^***^ |
| Note: This table reports the Breusch–Godfrey test statistics and Durbin–Watson values for the workplace and residential mobility OLS models, evaluating the presence of temporal autocorrelation in the residuals. ^***^p < 0.001, ^**^p < 0.01, ^*^p < 0.05 | | |
