# Supplementary material for: Bayesian spatio-temporal modeling for policy evaluation: Sensitivity of policy effect estimates in the context of COVID-19 stay-at-home orders
Source: PLoS One. 2026 Feb 10;21(2):e0339196. doi: 10.1371/journal.pone.0339196 (PMC12890128; doi:10.1371/journal.pone.0339196)
Supplement: S5 Table — Note: In Column (1), standard errors are reported in parentheses (***p < 0.001, **p < 0.01, *p < 0.05). Columns (2) – (4) present the posterior means of the estimated coefficients, with 95% Bayesian credible intervals shown in brackets. Posterior means marked with † indicate that the 95% credible interval does not include zero, signifying statistical significance. (DOCX) [file pone.0339196.s007.docx]

**Supporting Information**

**S5 Table. Full Results from Bayesian Spatial Model**

| Variable | | (1) Workplace Mobility | (2) Residential Mobility |
| --- | --- | --- | --- |
| Stay-at-home (recommended) | | -0.072^†^ [-0.082; -0.061] | 0.002 [-0.004; 0.008] |
| Stay-at-home (mandatory) | | -0.284^†^ [-0.297; -0.270] | 0.126^†^ [0.118; 0.133] |
| COVID-19 case (log) | | -0.958^†^ [-1.020; -0.895] | 0.455^†^ [0.420; 0.490] |
| Vaccination rate | | 4.592^†^ [3.893; 5.292] | -5.655^†^ [-6.028; -5.282] |
| Mask mandates | | 0.016^†^ [0.007; 0.025] | -0.048^†^ [-0.053; -0.042] |
| Public campaign | | -0.360^†^ [-0.376; -0.343] | 0.071^†^ [0.062; 0.081] |
| Economic support | | -3.049^†^ [-3.161; -2.937] | 1.638^†^ [1.573; 1.702] |
| Population density (log) | | 0.118 [-0.165; 0.400] | 0.246^†^ [0.117; 0.374] |
| Household size | | 0.411 [-0.542; 1.365] | 1.906^†^ [1.382; 2.431] |
| Non-white population share | | 2.555^†^ [0.992; 4.126] | -0.208 [-0.966; 0.548] |
| Unemployment rate | | -12.829^†^ [-21.171; -4.480] | -2.382 [-8.302; 3.536] |
| Share of population aged 65 and older | | 57.010^†^ [50.378; 63.646] | -18.649^†^ [-21.536; -15.754] |
| Share of population with a bachelor’s degree or higher | | -35.891^†^ [-41.001; -30.725] | 19.245^†^ [17.231; 21.253] |
| Intercept | | -13.700^†^ [-17.265; -10.142] | -2.645^†^ [-4.553; -0.741] |
| Precision Values for Random Effects | Gaussian Observations | 0.039^†^  [0.039; 0.04] | 0.225^†^  [0.219; 0.231] |
|  | County IID | 0.042^†^  [0.033; 0.049] | 0.491^†^  [0.436; 0.551] |
|  | County CAR (IID) | 12.8^†^  [0.969; 74.4] | 4354.859^†^  [10.528; 30500] |
|  | County CAR (Spatial) | 38100000^†^  [40.167; 24900000] | 1934.104^†^  [71.122; 6620] |
|  | Month AR (1) |  |  |
|  | ρ for Month AR (1) |  |  |
|  | Space Time IID |  |  |
| Model Fit | DIC | 136577.54 | 53558.22 |
|  | WAIC | 136628.84 | 53576.20 |
|  | MLL | -103643.09 | -61373.22 |
| Note: In Column (1), standard errors are reported in parentheses (^***^p < 0.001, ^**^p < 0.01, ^*^p < 0.05). Columns (2) – (4) present the posterior means of the estimated coefficients, with 95% Bayesian credible intervals shown in brackets. Posterior means marked with † indicate that the 95% credible interval does not include zero, signifying statistical significance. | | | |
