# Supplementary material for: Bayesian spatio-temporal modeling for policy evaluation: Sensitivity of policy effect estimates in the context of COVID-19 stay-at-home orders
Source: PLoS One. 2026 Feb 10;21(2):e0339196. doi: 10.1371/journal.pone.0339196 (PMC12890128; doi:10.1371/journal.pone.0339196)
Supplement: S6 Table — Note: In Column (1), standard errors are reported in parentheses (***p < 0.001, **p < 0.01, *p < 0.05). Columns (2) – (4) present the posterior means of the estimated coefficients, with 95% Bayesian credible intervals shown in brackets. Posterior means marked with † indicate that the 95% credible interval does not include zero, signifying statistical significance. (DOCX) [file pone.0339196.s008.docx]

**Supporting Information**

**S6 Table. Full Results from Bayesian Temporal Model**

| Variable | | (1) Workplace Mobility | (2) Residential Mobility |
| --- | --- | --- | --- |
| Stay-at-home (recommended) | | -0.013^†^ [-0.026; -0.001] | 0.007^†^ [0.002; 0.013] |
| Stay-at-home (mandatory) | | -0.052^†^ [-0.068; -0.036] | 0.012^†^ [0.005; 0.019] |
| COVID-19 case (log) | | -0.018 [-0.067; 0.031] | 0.149^†^ [0.126; 0.171] |
| Vaccination rate | | -15.297^†^ [-18.858; -11.738] | 3.884^†^ [2.246; 5.524] |
| Mask mandates | | -0.003 [-0.016; 0.010] | 0.029^†^ [0.023; 0.036] |
| Public campaign | | -0.001 [-0.025; 0.023] | 0.016^†^ [0.004; 0.028] |
| Economic support | | -0.081 [-0.238; 0.076] | 0.593^†^ [0.518; 0.667] |
| Population density (log) | | -0.462^†^ [-0.544; -0.380] | 0.460^†^ [0.417; 0.503] |
| Household size | | -3.031^†^ [-3.445; -2.618] | 2.311^†^ [2.109; 2.512] |
| Non-white population share | | -0.837^†^ [-1.284; -0.389] | 0.528^†^ [0.269; 0.786] |
| Unemployment rate | | -13.501^†^ [-16.869; -10.133] | -4.595^†^ [-6.925; -2.264] |
| Share of population aged 65 and older | | 40.653^†^ [38.507; 42.799] | -13.282^†^ [-14.287; -12.277] |
| Share of population with a bachelor’s degree or higher | | -51.626^†^ [-53.098; -50.153] | 20.586^†^ [19.928; 21.244] |
| Intercept | | -10.535^†^ [-14.584; -6.172] | -4.845^†^ [-7.061; -2.790] |
| Precision Values for Random Effects | Gaussian Observations | 0.034^†^  [0.034; 0.035] | 0.277^†^  [0.270; 0.288] |
|  | County IID |  |  |
|  | County CAR (IID) |  |  |
|  | County CAR (Spatial) |  |  |
|  | Month AR (1) | 0.033^†^  [0.021; 0.048] | 0.146^†^  [0.110; 0.227] |
|  | ρ for Month AR (1) | 0.385^†^  [0.127; 0.596] | 0.445^†^  [0.107; 0.681] |
|  | Space Time IID |  |  |
| Model Fit | DIC | 138252.62 | 50442.88 |
|  | WAIC | 138244.02 | 50445.92 |
|  | MLL | -69298.80 | -25362.33 |
| Note: In Column (1), standard errors are reported in parentheses (^***^p < 0.001, ^**^p < 0.01, ^*^p < 0.05). Columns (2) – (4) present the posterior means of the estimated coefficients, with 95% Bayesian credible intervals shown in brackets. Posterior means marked with † indicate that the 95% credible interval does not include zero, signifying statistical significance. | | | |
