# Supplementary material for: Bayesian spatio-temporal modeling for policy evaluation: Sensitivity of policy effect estimates in the context of COVID-19 stay-at-home orders
Source: PLoS One. 2026 Feb 10;21(2):e0339196. doi: 10.1371/journal.pone.0339196 (PMC12890128; doi:10.1371/journal.pone.0339196)
Supplement: S7 Table — Note: In Column (1), standard errors are reported in parentheses (***p < 0.001, **p < 0.01, *p < 0.05). Columns (2) – (4) present the posterior means of the estimated coefficients, with 95% Bayesian credible intervals shown in brackets. Posterior means marked with † indicate that the 95% credible interval does not include zero, signifying statistical significance. (DOCX) [file pone.0339196.s009.docx]

**Supporting Information**

**S7 Table. Full Results from Bayesian Spatio-Temporal Model**

| Variable | | (1) Workplace Mobility | (2) Residential Mobility |
| --- | --- | --- | --- |
| Stay-at-home (recommended) | | -0.171 [-6.104; 5.761] | 0.051 [-5.882; 5.983] |
| Stay-at-home (mandatory) | | -0.253 [-6.718; 6.212] | 0.077 [-6.388; 6.541] |
| COVID-19 case (log) | | -0.314 [-5.070; 4.442] | 0.190 [-4.566; 4.946] |
| Vaccination rate | | -4.756 [-13.421; 3.909] | 8.201^†^ [0.262; 16.139] |
| Mask mandates | | -0.061 [-6.329; 6.206] | 0.020 [-6.247; 6.288] |
| Public campaign | | 0.035^†^ [0.017; 0.054] | 0.019^†^ [0.009; 0.029] |
| Economic support | | -7.628^†^ [-8.415; -6.841] | 1.404^†^ [0.935; 1.870] |
| Population density (log) | | -0.542^†^ [-0.810; -0.276] | 0.565^†^ [0.446; 0.684] |
| Household size | | -0.388 [-1.149; 0.373] | 2.030^†^ [1.611; 2.448] |
| Non-white population share | | -2.769^†^ [-4.259; -1.287] | -0.503 [-1.204; 0.192] |
| Unemployment rate | | -6.525 [-13.441; 0.393] | 1.027 [-3.870; 5.934] |
| Share of population aged 65 and older | | 35.899^†^ [29.615; 42.157] | -13.278 [-15.888; -10.670] |
| Share of population with a bachelor’s degree or higher | | -40.465^†^ [-45.201; -35.702] | 20.282 [18.436; 22.122] |
| Intercept | | -10.572^†^ [-13.801; -7.342] | -6.434 [-8.144; -4.725] |
| Precision Values for Random Effects | Gaussian Observations | 0.386^†^  [0.092; 1.158] | 1.12^†^  [0.240; 3.20] |
|  | County IID | 0.046^†^  [0.042; 0.050] | 0.557^†^  [0.373; 0.720] |
|  | County CAR (IID) | 3278.69^†^  [687.21; 9975.17] | 92.9^†^  [1.162; 625.0] |
|  | County CAR (Spatial) | 994.442^†^  [106.324; 4115.429] | 181,000^†^  [77.916; 939,000] |
|  | Month AR (1) | 38.536^†^  [4.598; 137.693] | 1,140^†^  [0.716; 7,370] |
|  | ρ for Month AR (1) | 0.833^†^  [0.443; 0.974] | 0.261  [-0.948; 0.992] |
|  | Space Time IID | 0.113^†^  [0.059; 0.189] | 2.02^†^  [0.304; 7.45] |
| Model Fit | DIC | 108530.54 | 40032.33 |
|  | WAIC | 106465.55 | 41210.03 |
|  | MLL | -96362.93 | -56541.65 |
| Note: In Column (1), standard errors are reported in parentheses (^***^p < 0.001, ^**^p < 0.01, ^*^p < 0.05). Columns (2) – (4) present the posterior means of the estimated coefficients, with 95% Bayesian credible intervals shown in brackets. Posterior means marked with † indicate that the 95% credible interval does not include zero, signifying statistical significance. | | | |
