# Supplementary material for: Bayesian spatio-temporal modeling for policy evaluation: Sensitivity of policy effect estimates in the context of COVID-19 stay-at-home orders
Source: PLoS One. 2026 Feb 10;21(2):e0339196. doi: 10.1371/journal.pone.0339196 (PMC12890128; doi:10.1371/journal.pone.0339196)
Supplement: S8 Table — Note: As a sensitivity test, the models were re-estimated after excluding counties with more than 30% missing observations. In Column (1), standard errors are reported in parentheses (***p < 0.001, **p < 0.01, *p < 0.05). Columns (2) – (4) present the posterior means of the estimated coefficients, with 95% Bayesian credible intervals shown in brackets. Posterior means marked with † indicate that the 95% credible interval does not include zero, signifying statistical significance. (DOCX) [file pone.0339196.s010.docx]

**Supporting Information**

**S8 Table. Bayesian Spatio-Temporal Model Estimates After Excluding Counties with More Than 30% Missing Mobility Data**

| Variable | | (1) Workplace Mobility | (2) Residential Mobility |
| --- | --- | --- | --- |
| Stay-at-home (recommended) | | -0.096 [-6.029; 5.837] | 0.050 [-5.882; 5.983] |
| Stay-at-home (mandatory) | | -0.168 [-6.633; 6.297] | 0.077 [-6.388; 6.541] |
| COVID-19 case (log) | | -0.258 [-5.014; 4.498] | 0.175 [-4.581; 4.931] |
| Vaccination rate | | -5.651 [-14.787; 3.481] | 9.213^†^ [1.205; 17.221] |
| Mask mandates | | -0.073 [-6.341; 6.194] | 0.016 [-6.251; 6.284] |
| Public campaign | | 0.014 [-0.010; 0.037] | 0.019^†^ [0.009; 0.030] |
| Economic support | | -8.631^†^ [-9.606; -7.656] | 1.520^†^ [1.086; 1.955] |
| Population density (log) | | -1.412^†^ [-1.734; -1.091] | 0.511^†^ [0.383; 0.638] |
| Household size | | -0.457 [-1.524; 0.616] | 2.331^†^ [1.889; 2.773] |
| Non-white population share | | -2.091^†^ [-3.986; -0.204] | -0.409 [-1.176; 0.353] |
| Unemployment rate | | -0.555 [-11.833; 10.720] | -0.625 [-6.109; 4.869] |
| Share of population aged 65 and older | | 38.959^†^ [32.408; 45.495] | -13.866^†^ [-16.589; -11.146] |
| Share of population with a bachelor’s degree or higher | | -45.631^†^ [-50.279; -40.958] | 20.186^†^ [18.297; 22.071] |
| Intercept | | -5.243^†^ [-9.435; -1.061] | -6.434^†^ [-8.297; -4.725] |
| Precision Values for Random Effects | Gaussian Observations | 0.925^†^  [0.014; 5.990] | 4.88^†^  [0.191; 28.40] |
|  | County IID | 0.084^†^  [0.074; 0.094] | 0.588^†^  [0.356; 0.807] |
|  | County CAR (IID) | 5150.0^†^  [61.330; 32000.0] | 32.6^†^  [1.470; 211.0] |
|  | County CAR (Spatial) | 52,100.0^†^  [0.333; 193,000] | 1,460,000.0^†^  [0.009; 688,000] |
|  | Month AR (1) | 3,700.0^†^  [0.017; 12,400] | 2,780,000.0^†^  [0.031; 782,000] |
|  | ρ for Month AR (1) | 0.494  [-0.950; 1.000] | 0.499  [-0.944; 0.999] |
|  | Space Time IID | 0.365^†^  [0.013; 1.910] | 0.991^†^  [0.155; 3.080] |
| Model Fit | DIC | 56295.93 | 34057.64 |
|  | WAIC | 56230.37 | 33832.35 |
|  | MLL | -65015.02 | -54507.85 |
| Note: As a sensitivity test, the models were re-estimated after excluding counties with more than 30% missing observations. In Column (1), standard errors are reported in parentheses (^***^p < 0.001, ^**^p < 0.01, ^*^p < 0.05). Columns (2) – (4) present the posterior means of the estimated coefficients, with 95% Bayesian credible intervals shown in brackets. Posterior means marked with † indicate that the 95% credible interval does not include zero, signifying statistical significance. | | | |
